# Vascular alterations among male elite athletes recovering from SARS-CoV-2 infection

**DOI:** 10.1038/s41598-022-12734-z

**Published:** 2022-05-23

**Authors:** Pascal Bauer, Lutz Kraushaar, Oliver Dörr, Stanislav Keranov, Holger Nef, Christian W. Hamm, Astrid Most

**Affiliations:** 1 Department of Cardiology and Angiology, Justus- Liebig- University Giessen, 35390 Giessen, Germany; 2 Adiphea GmbH, Werbach, Germany; 3 Department of Cardiology, Kerckhoff Clinic GmbH, Bad Nauheim, Germany

**Keywords:** Circulation, Physiology, Diseases, Infectious diseases, Viral infection

## Abstract

SARS-CoV-2 may affect the cardiovascular system and vascular impairment has been reported in healthy young adults recovering from COVID-19. However, the impact of SARS-CoV-2 infection on the vascular function of elite athletes is unknown. We examined 30 healthy male elite athletes (age 25.8 ± 4.6 years) pre-season and at a 6-month follow-up (182 ± 10 days). Vascular function and central blood pressure were calculated using transfer function-based analysis of peripheral arterial waveforms obtained by oscillometry. We performed a two-way repeated-measures ANOVA on the biomarker data, with SARS-CoV-2 status as the between-groups factor and time as the within-groups factor. Subjects who tested positive for SARS-CoV-2 were studied 18 ± 4 days after their positive testing date at follow-up. Of 30 athletes, 15 tested positive for SARS-CoV-2 after the first examination and prior to the follow-up. None had severe COVID-19 or reported any persisting symptoms. The results of the two-way repeated measures ANOVA revealed that there was no significant main effect of COVID-19 on any of the investigated biomarkers. However, there was a significant interaction between the effects of SARS-CoV-2 exposure and time on augmentation index (Aix) (*p* = 0.006) and augmentation index normalized to a heart rate of 75 beats per minute (Aix@75), (*p* = 0.0018). The observation of an interaction effect on Aix and Aix@75 in the absence of any main effect indicates a cross-over interaction. Significant vascular alterations in male elite athletes recovering from COVID-19 were observed that suggest vascular impairment. Whether these alterations affect athletic performance should be evaluated in future studies.

## Introduction

Severe acute respiratory syndrome coronavirus 2 (SARS-CoV-2) induces the new coronavirus disease 2019 (COVID-19), which has rapidly evolved into an extensive pandemic. The virus leads to severe lung disease, with acute respiratory distress syndrome^1^ being its most prominent form, but many other organs such as the heart, kidney, brain and the vascular system may also be involved^2^. SARS-CoV-2 can infiltrate into human cells using the angiotensin-converting enzyme 2, a cell receptor found in various human tissues such as the lungs, heart, and blood vessels with different expression levels^3,4^. Cellular infection initiates localized inflammation, endothelial activation, tissue damage, and disordered release of cytokines^5,6^. Patients with cardiovascular diseases are thus at higher risk of severe SARS-CoV-2- associated complications and death^7^. SARS-CoV-2 itself, however, was shown to directly affect the cardiovascular system, leading to an increased risk of cardiovascular events, especially in men^8,9^, and endothelial dysfunction^10–12^. Even in healthy young individuals recovering from a SARS-CoV-2 infection, significant vascular alterations including increased arterial stiffness and decreased vascular function as assessed by flow-mediated dilation (FMD), were observed^13^, raising concerns about the long-term effects of the infection^14^. Further, another study^15^ reported higher carotid stiffness and aortic stiffness among young adults (mean age 20 years) recovering from SARS-Cov-2 compared to uninfected age-matched controls.

Though, the participants were non-athletes. Thus far, information concerning vascular alterations in elite athletes recovering from COVID-19 is not available. Moreover, longitudinal data about vascular functional parameters before and after SARS-CoV-2 infection are sparse.

We showed in our prior investigations using non-invasive oscillometric devices^16^ that elite athletes display lower PWV and an enhanced vascular function compared to the normal population^17^. We further investigated the association between vascular function, performance levels and maximum oxygen uptake^18^.

A higher cardiorespiratory fitness was associated with a lower risk for a severe COVID-19 course^19^ and long- COVID-19 syndrome and partly attributed to an enhanced vascular function^20^. Hence, both the exercise-induced adaptations of the cardiovascular system, leading to an enhanced vascular function compared to non-athletes^17^, and the high fitness level of elite athletes might mitigate the course of COVID-19 with reduction of the vascular impairment that is associated with the infection. Consequently, these individuals' vascular functional status may represent the strongest natural defense against the direct viral insults and the inflammatory sequelae of SARS-CoV-2 infection. Comparison of the responses of vascular functional biomarkers to SARS-CoV-2 infection in this “elite- athlete” model versus their non-infected peers may uncover sentinel markers-representing the proverbial weakest link in the complex chain of vascular function. Such biomarkers may serve as preventive and therapeutic targets in the general population.

Moreover, because vascular function directly impacts physical performance in elite athletes^18^, vascular impairment after SARS-CoV-2 infection might affect physical performance negatively.

Therefore, we investigated central hemodynamic parameters and vascular function in a cohort of male elite athletes at two time points and compared athletes recovering from COVID-19 with those who remained uninfected.

We hypothesized that SARS-CoV-2 infection would affect systemic arterial stiffness without impairing cardiovascular function at rest in athletes as this population’s training induced functional reserve would be able to compensate any increase in stiffness. Correspondingly we expected to see no significant differences in functional parameters between non-infected athletes and their peers recovering from infection.

## Methods

### Study design

The study was conducted as a cross-sectional, single-center pilot study. All athletes included were first examined pre-season in July and August 2020 (T1) after a six-week competition-free interval. The follow- up examination was performed during the competition season between December 2020 and January 2021 (T2).

The handball season started at October 1st and the regular ice hockey season was initially planned to start at October 2nd. Due to COVID-19 restrictions, the season started at the end of October. The second German soccer league started September 18th.

Hence, pre-season preparation started end of July in handball, mid-July in soccer and mid-August in ice-hockey.

Due to the outbreak of COVID-19 in the respective professional teams, all identified COVID-19 contact- team members and staff were ordered to quarantine for 14 days by health care government, irrespective of symptoms or positive tests. The COVID-19 outbreaks took place at similar time points (November and December). None of the included 30 athletes exercised during the quarantine period of 14 days before the second examination (T2) was performed. T2 was part of the mandatory return-to-sports algorithm after COVID-19 of the respective professional sports-leagues.

All participants provided their written informed consent. Further, they filled out a questionnaire regarding health status, medication, nutrition supplementation, amount of training, and history of training. Only individuals free of underlying cardiovascular diseases, risk factors, and medication were included. All tests were conducted at least 3 h post- prandially, and subjects refrained from exercise for at least 36 h prior to the test. There was no restriction of caffeine intake provided. Thus, alcohol consumption was prohibited the two days prior to the examinations.

The local ethics committee of the University of Giessen approved the study protocol (AZ183/20). The study was performed in accordance with the ethical standards laid down in the Declaration of Helsinki and its later amendments.

### Study population

The participants were 30 male professional athletes, 15 of whom contracted COVID-19 during the follow-up period. Fifteen athletes remained uninfected with SARS-CoV-2 at both initial (T1) and follow-up (T2) examinations.

The 30 healthy professional athletes consisted of 15 handball, 14 ice-hockey and 1 soccer player of the first German handball division (Bundesliga), second German ice hockey (DEL2) and soccer (2. Bundesliga) league, respectively.

Seven ice-hockey and 7 handball players as well as 1 soccer player got infected during the study period, whereas 8 handball and 7 ice-hockey players remained uninfected.

All participants included were Caucasian non-smokers and none took medication or multivitamin supplements.

Athletes were included in the SARS-CoV-2 group if they tested positive for SARS-CoV-2 using a nasopharyngeal swab and the polymerase chain reaction assay. They were only included if the infection occurred after the first and prior to the follow-up examination. The control group consisted of athletes who completed both examinations and tested negative for SARS-CoV-2 using the abovementioned PCR test at both time points. In addition, all athletes included tested negative for SARS-CoV-2 antibodies at the first examination.

In addition, all athletes received SARS-CoV-2 antibody tests at the follow-up examination (T2). The respective blood tests for IgM and IgG class anti-SARS-CoV-2 antibodies were performed using the m2000 SARS-CoV-2 assay (Abbott Laboratories, Chicago, Illinois, USA). The assay is a chemiluminescent immunoassay that detects IgM and IgG raised against the nucleocapsid protein of SARS-CoV-2. A signal/cut-off (S/CO) ratio of ≥ 1.4 was interpreted as reactive and an S/CO ratio of < 1.4 was interpreted as non-reactive.

All individuals were subjected to a physical examination and 12-lead electrocardiogram (ECG). Age, height, weight, and body mass index were determined at the first examination. Weight and height were measured with a validated commercial combination scale to weigh and to determine height (Seca GmbH &Co Ag, Hamburg, Germany). Body surface area was calculated using the formula of DuBois^21^. Blood pressure (BP) measurements and non- invasive vascular examinations were performed, as described below.

### Non-invasive assessment of peripheral and central blood pressure and pulse pressure waveforms

We used the non-invasive vascassist2® device (isymed GmbH, Butzbach, Germany) to acquire pulse pressure waveforms by means of oscillometry. The device uses a validated model^22,23^ of the arterial tree that consists of 721 electronic circuits representing all central and peripheral arterial sections. By modulating the circuits’ capacitance, resistance, inductance, and voltage, the system replicates an individual’s acquired pulse pressure waves. The vascassist2® system is currently unique in the use of genetic algorithms to optimize the fidelity of the pulse pressure wave replication^22^. Fidelity replications of 99.6% or above were included in the analysis.

The non-invasive vascular evaluation was carried out for all participants after a 15-min rest period. Measurements were performed in a supine position using four conventional cuffs adapted to the upper arm and forearm circumferences of the participants. Radial and brachial pulse pressure waves were acquired on both arms with step-by-step deflation of the cuffs. The measurements took place in a room with a comfortable and stable temperature of 22 °C and a lack of external stress influences. Participants were advised not to move during the acquisition of pulse pressure waves. Two brachial and three radial measurements were performed to guarantee stable and valid results with a break of 30 s between each measurement phase. The total duration of the examination was 15 min. The acquired pulse pressure waves were then analyzed with a validated electronic model of the arterial tree to assess vascular functional parameters. Brachial and radial systolic (SBP) and diastolic (DBP) BP, central systolic and diastolic BP (CBP), aortic pulse wave velocity (PWV), augmentation index (Aix), augmentation index at a heart rate of 75 bpm (Aix@75), resistance index (R), total vascular resistance, and ejection duration were calculated. CBP was determined using a validated transfer function that was based on the peripheral arterial waveform^23^. Calculation of Aix@75 was also based on the pulse waveform.

### Statistical analysis

Descriptive analyses were carried out on all study variables for the total sample and separated by SARS-CoV-2 infection status. All data are presented as mean ± standard deviation (SD). The Shapiro–Wilk test was used to determine normality of distribution. If the data were determined to have a skewed distribution, all analyses were performed on normalized data after appropriate conversion.

A two-way repeated measures ANOVA was conducted on a sample of 60 athletes with group (SARS-CoV-2 and controls) as the between factor and time (T1 and T2) as the within factor to examine the effect of SARS-CoV-2 exposure on Aix and Aix@75. Bonferroni correction for multiple testing were performed. As four comparisons were made using the same data (SARS-CoV-2 at T1 vs. Controls at T1, SARS-CoV-2 at T2 vs. Controls at T2, SARS-CoV-2 at T1 vs. SARS-CoV-2 at T2, Controls at T1 vs. Controls at T2) correlation analyses on the same dependent variable indicated the need for a Bonferroni correction of (αaltered = 0.05/4) = 0.0125. Hence, the statistical significance was set at *p* < 0.0125 (two-tailed) for all measurements.

All statistical analyses were performed using the statistical software IBM SPSS Statistics for Macintosh, Version 25.0 (IBM Corp., Armonk, NY, USA).

## Results

### Cohort characteristics

All 30 male athletes included in the study were participants in mixed team sports disciplines that are characterized by a high- intensity level (handball, ice- hockey, and soccer)^24^. The mean age of the participants was 25.8 ± 4.6 years with a mean height of 188.5 ± 6.7 cm and a mean weight of 93.1 ± 8.4 kg, resulting in mean body mass index of 26.2 ± 1.8 kg/m^2^. The probands were experienced athletes who had participated in professional training for 10 ± 4.6 years with a current mean training time of 19.6 ± 2 h per week.

There were no significant differences regarding age, height, weight, body mass index, body surface area, training history and amount of training per week between athletes who tested positive for SARS-CoV-2 after the baseline examination and those who remained uninfected. The clinical characteristics, anthropometric data, and specific training data are displayed in detail in Table 1.

**Table 1:** Clinical characteristics of the athletes included at the first examination.

	SARS-CoV-2 positive		Controls		*p* value
	n = 15		n = 15		
	Mean	SD	Mean	SD	
Age (years)	26	4	25.6	5.3	0.847
Height (cm)	187.1	6.9	189.9	6.3	0.254
Weight (kg)	92.6	8.1	93.7	9.1	0.736
Body mass index (kg/m^2^)	26.4	1.3	26	2.1	0.491
Body surface area (m^2^)	2.18	0.13	2.21	0.12	0.485
Training history (years)	9.87	4.2	10.13	5.2	0.878
Training per week (h)	20.1	1.6	19	2.3	0.109

The follow-up examinations (T2) were performed 182 ± 10 days after the first examinations (T1) with no difference (*p* > 0.05) between athletes recovering from SARS-CoV-2 infection (181 ± 14 days) and controls (188 ± 12 days).

Subjects who tested positive for SARS-CoV-2 were studied 18 ± 4 days after their positive testing date at T2. Five of the 15 athletes who tested positive had remained asymptomatic throughout the quarantine period of 14 days. Nine athletes displayed mild symptoms for 3–5 days and one athlete described mild symptoms for 8 days. None of the infected athletes were hospitalized or required medical therapy. At T2, all athletes were free of symptoms.

### Blood pressure and vascular function at baseline and follow-up measurement

All participants displayed a brachial BP below 140/90 mmHg for both measurements.

All biomarker data were normally distributed, with the exception pf PWV. While inverting the PWV data restored normality of distribution, it did not materially alter the results, with neither main effects nor interaction effects becoming significant.

### Differences between baseline and follow-up measurements in the two groups

The results of the two-way mixed ANOVA showed that there were no significant main effects of SARS-CoV-2 infection on any of the investigated biomarkers (Table 2). In contrast, we observed a significant main effect of time on peripheral and aortic systolic and diastolic blood pressure as well as on aortic mean blood pressure, with all markers showing an increase from baseline to follow-up. Detailed data are given in Table 2.

**Table 2:** Results of vascular evaluation showing differences between the baseline (T1) and follow-up examination (T2) in SARS-CoV-2 positive athletes (n = 15) and controls (n = 15).

	SARS-CoV-2				Controls				p	p	p
	T1		T2		T1		T2				
	Mean	SD	Mean	SD	Mean	SD	Mean	SD	COVID	Time	CxT
Brachial systolic BP (mmHg)	126	9.2	128	5.1	126	7.1	128	8	0.83	0.2	0.89
Brachial diastolic BP (mmHg)	62	7.5	66.3	8.4	59	8.4	64.9	7.3	0.41	< **0.01**	0.81
Mean brachial BP (mmHg)	77.6	6.5	84.3	7.1	75.3	6.1	81.7	4.7	0.15	< **0.001**	0.93
Pulse pressure (mmHg)	64.1	11	61.4	13.1	65.6	11.2	63.4	10.6	0.52	0.12	0.91
Heart rate at rest (bpm)	54.8	8.8	59.3	8.6	62.1	12.4	60.1	11.7	0.22	0.53	0.12
Mean aortic BP (mmHg)	74.8	7.3	78.8	7.2	74.7	7.4	79.6	7.8	0.87	< **0.01**	0.77
Central systolic BP (mmHg)	99	6.3	101.5	4.8	98.6	5	102.5	4.7	0.88	**0.008**	0.42
Central diastolic BP (mmHg)	60.8	8.9	65.4	9.2	59.2	8.4	64.8	7.2	0.68	< **0.01**	0.76
Aortic augmentation pressure (mmHg)	− 6.3	3.8	− 4.6	2.9	− 5.9	4.7	− 4.5	4.3	0.81	0.07	0.87
Aortic pulse wave velocity (m/s)	6	0.74	6.3	0.81	6.1	1.1	6.1	0.66	0.91	0.22	0.31
Augmentation index (%)	− 16.1	9.4	− 11.2	8.5	− 15.8	10.7	− 20	5.4	0.20	0.83	**0.006**
Augmentation index @75 (%)	− 26	8.2	− 20.5	8.2	− 20.5	13.3	− 23.6	6	0.68	0.46	**0.002**
Total vascular resistance (dyn*s/cm^5^)	1436	424	1404	449	1288	259	1275	250	0.24	0.70	0.87
Ig-M SARS CoV-2 (S/CO)	0		6.1	2.1	0		0		< **0.001**		
Ig-G SARS CoV-2 (S/CO)	0		4.2	2.9	0		0		< **0.001**		

Bold values denote statistical significance at the p< 0.0125 level.

C/T, COVID by time interaction effect; *p* values are for main effects (COVID and Time respectively) and for interaction effect; BP, Blood pressure; S/CO, Signal/cutoff ratio.

There was a significant interaction between the effects of COVID exposure and time on augmentation index (F(1, 28) = 8.72, *p* = 0.006) and augmentation index normalized to a heart rate of 75 beats per minute, (F(1, 28) = 6.22, *p* = 0.0018). Significance remained unchanged after Huynh–Feldt, Greenhouse–Geisser and Box’s conservative F adjustment for violation of compound symmetry assumption.

**Figure 1:**
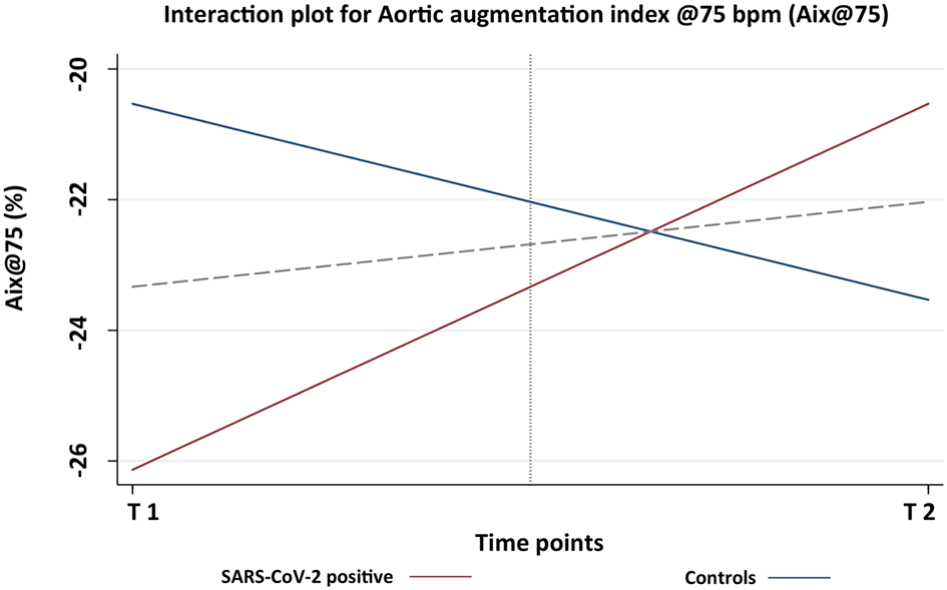
Aortic augmentation index @75 bpm at the two time points according to SARS-CoV-2 status and graphical depiction of the two-way repeated measures ANOVA.

The observation of an interaction effect on augmentation index in the absence of any main effect indicates a cross-over interaction. We display the results of the analysis for Aix@75 in Fig. 1. That is, the effect of time on the augmentation index is opposite depending on whether an infection with SARS-CoV-2 has occurred between measurement time points. All reported significances correspond to the applied Bonferroni correction for the 2-by-2 ANOVA design.

## Discussion

The present study, to our knowledge, represents the first detailed comparison of vascular alterations in male elite athletes recovering from SARS-CoV-2 infection versus their uninfected peers.

Our most important findings are that

1. athletes recovering from COVID-19 displayed an increase in Aix and Aix@75 that, in itself was not significant, but was significantly different from the (non-significant) decrease of Aix and Aix@75 observed in the control group; representing a significant cross-over interaction;
2. there were no significant interaction effects on any of the other vascular biomarkers;
3. however, there were significant main effects of time on peripheral and aortic blood pressure values which increased from baseline to follow-up.

The findings of increased Aix secondary to COVID-19 need to be viewed in the context of what determines Aix. The latter is mainly determined by the reservoir function of the most proximal segment of the aorta^25^. This segment is chiefly responsible for the cushioning of the systolic rise in blood pressure when the left ventricle ejects the blood bolus, and for the cushioning of the diastolic blood pressure fall when the aortic recoil maintains continued flow throughout the arterial system^25^.

Our observation of a significant increase in Aix suggests that SARS-CoV-2 infection may detrimentally affect aortic reservoir function. To which intent this functional deterioration impacts athletic performance warrants further investigation.

That we were able to observe this functional decline despite the excellent fitness levels of the participants and the mild course of the COVID-19 cases, hints at Aix as potential sentinel marker for the severity of vascular and endothelial involvement in COVID-19. As this index is influenced by heart rate, indexing for heart rate 75 is recommended^26^. Even after this adjustment, the significant alterations remained. The Aix@75 correlates with fitness levels^27,28^ and is characterized by negative values in highly trained athletes^27,29^, reflecting “supra-physiologic” cardiovascular properties and thus facilitating an enhanced athletic performance. In contrast, lower physical activity levels are associated to higher Aix@75 values^30^.

Notably, the uninfected athletes displayed lower values in the follow-up examination whereas the SARS-CoV-2 infected athletes showed a significant increase in Aix@75. Though there was no overall effect of either SARS-CoV-2 or time point (T1/T2) on Aix@75, we clearly demonstrated a typical cross- over interaction as illustrated in Fig. 1. The dashed grey line represents the main effect means for time, whereas the intercepts of the vertical dotted grey line with the slopes for the two exposure groups represent the main effect means for exposure. While the slope of the dashed line and the distance between the two intercepts of the dotted line are non-significant, the difference between the slopes of the two groups is clearly significant.

The suitability of Aix as a sentinel marker and benchmark for preventive and therapeutic interventions efficacy is underscored by observations that Aix@75 is quickly altered by increasing^31^ physical activity, and that it is linked to cardiorespiratory fitness^27^. An inverse correlation between Aix and cardiorespiratory fitness has not only been confirmed to hold in young adults at comparable age to our athletes^28^, but more generally in men until the 6th decade of life^32^. Taken together these observations suggest that our infected athletes’ functional Aix reserves, though negatively impacted relative to pre-infection, serve as buffer against the potentially detrimental effects of SARS-CoV- 2 infection on endothelial function. Though, our findings may be linked to impaired athletic performance in the SARS-CoV-2 group. However, this hypothesis has to be investigated in further studies.

We have to acknowledge that all included athletes had not exercised for 14 days due to quarantine, which might have influenced the results of our study^33^. After COVID-19, enhanced sympathetic neural activity was reported^34^, but was not associated with an increased heart rate in a normal population. In line with this, the detected increase in heart rate in the SARS-CoV-2 group was not statistically significant. Further, training cessation has been shown to increase resting heart rate in athletes^35^, which might additionally be taken into account.

Interestingly, Ratchford et al.^13^ also found significant vascular alterations with impaired vascular function, measured by FMD, in young and otherwise healthy individuals (mean age 23 years) recovering from COVID-19. However, reactive hyperaemia measurements in this study were unchanged, which was explained by differences in the pathophysiological pathways addressed^13^. The virus elicits an inflammatory response in endothelial cells that diminishes NO bioavailability^13^, resulting in endothelial dysfunction. Thus, COVID-19 is recognized as an endothelial disease^10^ with an important impact on the vasculature^11^. Consequently, the virus infection primarily influences the NO- dependent testing methods of vascular function such as FMD and Aix@75^10,13^. Hence, despite the different methods used to measure vascular function, the agreement between the findings of our study and those of Ratchford et al.^13^ clearly indicate mild vascular impairment in individuals recovering from COVID-19.

The aortic pulse wave velocity (PWV) is another acknowledged and well-validated marker of arterial stiffness and cardiovascular risk^36^. It is influenced by heart rate and blood pressure. A recently published study^37^ investigated the relationship between heart rate, pulse wave velocity and blood pressure. The authors stated that the significant effect of heart rate on PWV is expected to be evident only in subjects with increased arterial stiffness (PWV > 8.6 m/s) and older individuals with elevated blood pressure. As none of these factors were applicable in our study of young normotensive elite athletes with low arterial stiffness, potential confounding effects of heart rate and blood pressure on PWV were minimized.

In contrast to the aforementioned study^13^, we did not detect significant alterations in PWV in our study. Differences in the study cohorts might explain this finding, as we examined highly trained athletes with an enhanced vascular function who display a markedly lower PWV than the normal population^13,28^. Further, the values for PWV we determined are in line with those reported by other studies that investigated athletes^28,38,39^ and with those published in recent meta-analyses^31^. That we did not detect alterations in PWV but in Aix@75 suggests that Aix@75 may be a more sensitive marker than PWV for the detection of an early cardiovascular impairment in athletes in this setting. This suggestion finds support in the observation that endothelial NO pathway dependent function directly correlates with Aix in a dose–response relationship^40^.

The continuous high-intensity exercise training load might have caused the slight increase in central systolic BP detected, which was also observed by other researchers in athletes following high-intensity exercise training^31,41^. Our first measurement took place in a pre-season monitoring program after a six-week competition-free interval. The observation of in-season increased central blood pressure parameters in the uninfected athletes relative to off-season related lower training volume corresponds with Tomoto et al.’s observation of an elevation of aortic systolic BP in elite endurance athletes following a greater-than-normal training volume^42^.

Hence, the central systolic BP measured at follow-up, though significantly altered compared with baseline in the uninfected group, is far lower than the reference values reported by Herbert et al.^43^ for the age-adjusted normal population and, in addition, lower than the values reported for high-level dynamic sports athletes^41^.

The excellent cardiorespiratory fitness and vascular function of the participants may have affected the course of the SARS-CoV-2 infection^19^, as all athletes displayed none or only mild symptoms during infection and had no symptoms at the follow-up measurement, which was conducted 18 days after the first positive PCR- test. Thus, an enhanced fitness level seems to be protective against a severe disease course^19^, which was also reported by other researchers^44^.

In summary, all parameters measured in our study were in the normal range for all athletes. Whether the vascular alterations detected in athletes recovering from COVID-19 constitute a clinically relevant change that affects their athletic performance in the future has yet to be determined. Thus, because of the higher fitness levels and the enhanced cardiovascular functions of elite athletes compared with the normal population, we were able to detect even mild vascular impairment following SARS-CoV-2 infection. We suggest that the measurement of vascular functional parameters may be more sensitive compared to a morphological assessment to evaluate vascular abnormalities occurring in the acute phase after SARS-CoV-2 infection.

### Limitations and strengths

Our study has a few limitations. The number of participants limited its statistical power to reveal other associations or to determine diagnostic thresholds. The focus on Caucasian male elite mixed-sports athletes may limit extrapolation of the results to other ethnicities, other sport disciplines, to an older age group, or to women. Further, we did not control for hydration status, diet, especially not for caffeine uptake, and body composition. However, we included male elite athletes of the same age without cardiovascular disease and free of medication, and we controlled for confounders like physical inactivity. Moreover, we excluded pre-existing SARS-CoV-2 infection. The rigid design of measuring cardiovascular function, the stringent examination time points in both groups and the comparison to the uninfected control group must be mentioned; therefore, our cohort of elite male athletes, although small, was homogeneous, which strengthens our analysis.

## Conclusion

In our cohort of male elite athletes, we detected significant alterations of the Aix and Aix@75 in athletes recovering from COVID-19 compared with those who remained uninfected. This observation suggests a mild vascular impairment that may potentially lead to reduced athletic performance. Equally importantly, the detected deterioration of Aix and Aix@75 in the elite athletic model of optimal vascular function suggest Aix and Aix@75 to serve as potential sentinel markers and benchmark for preventive and therapeutic interventions in the context of SARS-CoV-2 infections. Both hypotheses should be evaluated in future studies.

## Abbreviations

ANOVA: Analysis of variance
Aix: Augmentation index
Aix@75: Augmentation index at a heart rate of 75 bpm
BP: Blood pressure
CBP: Central blood pressure
DBP: Diastolic blood pressure
ECG: Electrocardiogram
FMD: Flow-mediated dilation
PWV: Aortic pulse wave velocity
SARS-CoV-2: Severe acute respiratory syndrome coronavirus 2
SBP: Systolic blood pressure
S/CO: Signal/cutoff ratio
SD: Standard deviation
sPAP: Systolic pulmonary artery pressure
TAPSE: Tricuspid annular plane systolic excursion
